# D-Serine made by serine racemase in *Drosophila* intestine plays a physiological role in sleep

**DOI:** 10.1038/s41467-019-09544-9

**Published:** 2019-05-07

**Authors:** Xihuimin Dai, Enxing Zhou, Wei Yang, Xiaohui Zhang, Wenxia Zhang, Yi Rao

**Affiliations:** 1 PKU-IDG/McGovern Institute for Brain Research, Peking University, Beijing, 100871 China; 2 College of Life Sciences, Beijing Normal University, Beijing, 100875 China; 3 State Key Laboratory of Natural and Biomimetic Drugs, Peking University, Beijing, 100191 China; 4 Chinese Institute for Brain Research, Beijing, 102206 China

**Keywords:** Genetics, Neuroscience

## Abstract

Natural D-serine (D-Ser) has been detected in animals more than two decades ago, but little is known about the physiological functions of D-Ser. Here we reveal sleep regulation by endogenous D-Ser. Sleep was decreased in mutants defective in D-Ser synthesis or its receptor the N-methyl-D-aspartic receptor 1 (NMDAR1), but increased in mutants defective in D-Ser degradation. D-Ser but not L-Ser rescued the phenotype of mutants lacking serine racemase (SR), the key enzyme for D-Ser synthesis. Pharmacological and triple gene knockout experiments indicate that D-Ser functions upstream of NMDAR1. Expression of SR was detected in both the nervous system and the intestines. Strikingly, reintroduction of SR into specific intestinal epithelial cells rescued the sleep phenotype of *sr* mutants. Our results have established a novel physiological function for endogenous D-Ser and a surprising role for intestinal cells.

## Abstract (web summary)

The physiological function of endogenous D-serine remains a mystery. Here the authors show that endogenous D-serine plays an important role in regulating sleep and that, while the D-serine synthesizing enzyme serine racemase (SR) is expressed both in the nervous system and the intestines, the SR in the intestine is shown to be functionally sufficient for sleep regulation.

## Introduction

Amino acids exist in stereoisomers, with all common amino acids except glycine having L- and D-enantiomers depending on the relative spatial arrangement surrounding the α-carbon. Though L-amino acids were traditionally thought to be the only natural form, D-amino acids have been found in biological organisms. Free D-serine (D-Ser) has been found in species ranging from bacteria to mammals^1–4^. D-Ser is an effective co-agonist of the N-methyl-D-aspartate subtype of glutamate receptor (NMDAR)^5,6^. D-Ser is synthesized from L-Ser by serine racemase (SR)^7^ and degraded by D-amino acid oxidase (DAAO)^4^ and SR^8^. Distribution of D-Ser and NMDAR as determined by chemical measurement^9^ and immunohistochemistry^10^ supports D-Ser as an endogenous coagonist acting on the glycine modulatory site of the NR1 subunits of the NMDAR^11,12^. A role for endogenous D-Ser in synaptic transmission was confirmed by selective degradation of D-Ser with DAAO which attenuated NMDAR function and its rescue by D-Ser^13^. It was proposed that the synaptic NMDAR is activated by D-Ser, whereas the extrasynaptic NMDAR is gated by glycine^14^.

Sleep is important for animals and is regulated by both circadian and homeostatic processes^15^. While significant progress has been made in the molecular understanding of circadian rhythm, much less is known about homeostatic regulation of sleep. For more than a decade, *Drosophila* has been used as a model for genetic studies of sleep^16,17^. Genes and brain regions regulating sleep have been identified^18–21^.

Recently, NMDAR and D-Ser have been indicated to participate in sleep regulation in both flies and mammals^22–24^. However, whether D-Ser regulates sleep remains unclear. Here, through a genetic screen followed by a thorough investigation of the synthases, the oxidases, and the receptor of D-Ser, combined with pharmacological genetic epistasis experiments, we report evidence that sleep is regulated by D-Ser through NMDAR1. Furthermore, the synthases, the oxidases, and the receptor of D-Ser have all been found to be expressed in the central nervous system and in the intestine. Strikingly, the intestinal but not neuronal expression has been proved to be important for sleep regulation, indicating a novel role of the intestine in sleep regulation. Taken together, these results suggest that D-Ser made by intestinal SR promotes sleep through NMDAR1 in *Drosophila*.

## Results

### Decreased sleep in *shmt* mutants and rescue by L-Ser or D-Ser

**Fig. 1:**
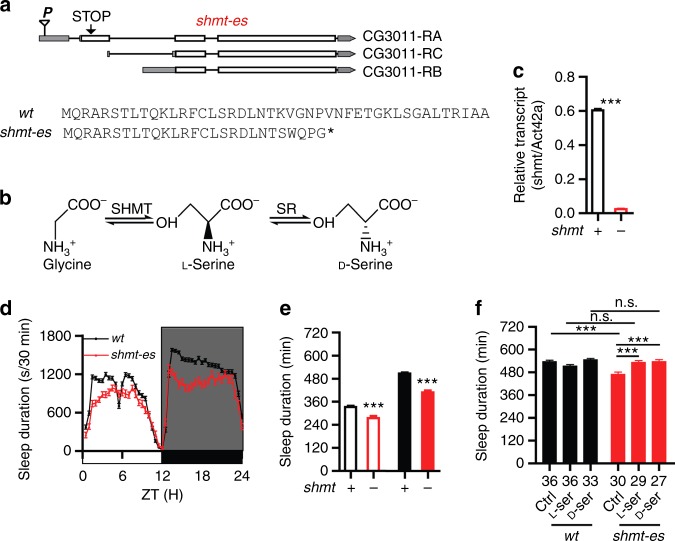
Sleep phenotypes of *shmt* mutants. **a** A schematic representation of a point mutation leading to a premature stop codon in *shmt* (thus *shmt-early stop* or *shmt-es*). Also shown is the amino acid sequences of the *shmt-es* mutant line used here. Single gRNA generated insertion and/or deletion (indel) in the *shmt* gene, introducing a frameshift and a stop codon (asterisk). **b** A diagram of D-Ser synthesis pathway. **c** mRNA level of isoform A *shmt* in *shmt-es* was significantly reduced. **d** Sleep profiles of *shmt-es* (red) (*n* = 57) and *wt* (black) (*n* = 236) flies, plotted in 30 min bins. White background indicates the light phase (ZT 0–12); shaded background indicates the dark phase (ZT 12–24). **e** Statistical analyses. Daytime and nighttime sleep durations were significantly reduced in *shmt-es* flies. In this and other figures, open bars denote daytime sleep and filled bars nighttime sleep. **f** Drug treatment of both L- and D-Ser rescued the nighttime sleep duration of *shmt-es* flies to the *wt* level. The number of flies used in the experiment was denoted under each bar. ***P < 0.001, n.s. *P* > 0.05, Mann–Whitney test was used in (**c**, **e**), two-way ANOVA test with Bonferroni posttests was used in (**f**) to compare the sleep durations between *wt* and *shmt-es*, Kruskal–Wallis test with Dunn’s posttest was used in (**f**) to compare the sleep durations of *shmt-es* under different drug treatments. Error bars represent s.e.m. Male flies were used

In a screen of homozygous P-element insertion lines for mutations affecting sleep, we found that sleep duration was decreased when a P element was inserted into the *CG3011* gene. Analysis of its sequence (Fig. 1a and Supplementary Fig. 1) indicates that *CG3011* encodes the serine hydroxymethyltransferase (SHMT), which participates in the synthesis of L-Ser^25,26^ (Fig. 1b). There are three isoforms of *shmt* in fly, the original mutant uncovered by our screen contained a P element insertion in the 5′ non-coding region of isoform A (Fig. 1a). To investigate the function of *Drosophila* SHMT, we generated mutations in the *shmt* gene by using CRISPR-Cas9. Deletion of all three isoforms caused lethality, whereas frameshift mutations introducing a STOP codon in the first coding exon of *shmt* affecting only isoform A resulted in viable *shmt* mutants (*shmt-es* in Fig. 1a). The mRNA level of isoform A *shmt* in *shmt-es* was significantly decreased compared with wild type (*wt*) flies detected by quantitative polymerase chain reaction (qPCR) analyses (Fig. 1c). The *shmt-es* mutants were backcrossed into an isogenic Canton-S (CS) line in our lab^27^, and used in further analysis.

Sleep was measured in *wt* and *shmt-es* flies by video recording and analysis. When tested under the 12 h light/12 h dark (LD) condition, durations of both nighttime sleep and daytime sleep were significantly decreased in *shmt-es* flies (Fig. 1d, e). Because L-Ser is the substrate for D-Ser synthesis (Fig. 1b)^7^, we tested whether the sleep phenotype of *shmt* mutants was attributed to L- or D-Ser by rescuing *shmt* mutants with either L-Ser or D-Ser. After eclosion, flies were raised with either sucrose or sucrose supplemented with L-Ser or D-Ser for 3 days before being transferred into recording tubes with the same media. Feeding either L-Ser or D-Ser could rescue the sleep defect of *shmt-es* flies (Fig. 1f). Thus, the sleep defect of *shmt-es* flies could be due to the lack of either L- or D-Ser.

### Decreased sleep and increased arousal in *sr* mutants

**Fig. 2:**
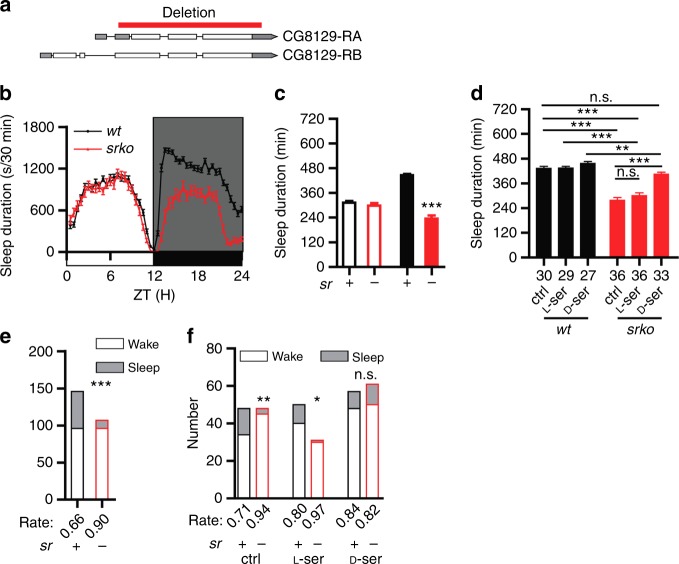
Sleep phenotype of *sr* mutants. **a** A schematic representation of the *CG8129* gene with the red bar indicating the region deleted in *srko* mutants. Two transcription variants (NM_141629, NM_169273) generate two proteins of 469aa (NP_649886) and 316aa (NP_731340), respectively. They have an identical C-terminal part while the longer variant has additional 153aa at the N-terminal region. In *srko*, aa 114–469 in the longer form and aa 1–316 in the shorter form were deleted. **b** Sleep profiles of *srko* (red) (*n* = 42) and *wt* (black) (*n* = 69) flies, plotted in 30 min bins. **c** Statistical analyses. Nighttime sleep durations were significantly reduced in *shmt-es* flies. **d** Drug treatment of D-Ser, but not L-Ser, rescued the nighttime sleep duration of *srko* flies to *wt* flies fed with mock. The number of flies used in the experiment was denoted under each bar. **e** Arousal rates of *srko* and *wt* flies under light stimuli. The arousal rate of *srko* flies was significantly increased. Numbers of flies that were aroused by the stimuli (open bars) and that kept sleep (filled bars) were plotted. Light stimuli were applied to *wt* and mutant flies as indicated. Arousal rate was denoted under each bar. **f** Drug treatment of D-Ser, but not L-Ser, rescued the arousal rate of *srko* flies to the *wt* level. ***P < 0.001, **P < 0.01, *P < 0.05, n.s. *P* > 0.05. Mann–Whitney test was used in (**c**), two-way ANOVA test with Bonferroni posttests was used in (**d**) to compare the sleep durations between *wt* and *srko* under the same treatment, Kruskal–Wallis test with Dunn’s posttest was used in (**d**) for other statistical analyses. Fisher’s exact test was used in (**e**, **f**). Error bars represent s.e.m. Male flies were used

SR is responsible for D-Ser production in vivo^28–30^. *Drosophila* SR is encoded by *CG8129* (Supplementary Fig. 2)^31^. To investigate the function of D-Ser, we generated *sr knock-out (srko)* flies in which most of the coding region of *sr* was deleted (Fig. 2a). Under LD condition, the nighttime sleep duration was significantly reduced in *srko* flies (Fig. 2b, c). We also generated four other *sr* mutants, including two deletion mutants *sr-middle* and *sr-long* (Supplementary Fig. 3a), and two insertion mutants *SRKO-Gal4* and *SRKO-Flp* with the coding region replaced by the yeast *Gal4* or *Flp* gene (Supplementary Fig. 3b). The duration of nighttime sleep but not that of daytime sleep was also reduced in these four mutants (Supplementary Fig. 3c-f). Because the nighttime sleep duration was decreased in all five *sr* mutants as well as the *shmt-es* mutants, we thereafter focused on the role of D-Ser in nighttime sleep, but not the daytime sleep which was observed in only the *shmt-es* mutants but none of the five *sr* deletion mutants.

While sleep duration can directly reflect sleep defect, stimuli-induced arousal rate can reflect sleep intensity. Previous studies have shown that sleep duration and arousal can be regulated separately^32,33^. We tested arousal response to light in *wt* and *srko* flies using similar method as previous studies^34^. On the 4th night after being transferred to the recording tubes, sleeping flies were shined with light pulses for 1 s at zeitgeber time (ZT) 16, and then the numbers of flies that were awaken and that kept sleep were counted. The arousal rate was significantly elevated in *srko* flies under stimulus (Fig. 2e).

Latency to sleep was increased in *srko* flies whereas circadian rhythm and sleep recovery after sleep deprivation were not significantly different between *srko* and wt flies (Supplementary Fig. 4). Taken together, these results indicate that *sr* is required for regulation of nighttime sleep and stimulus-induced arousal response.

### Role of D-Ser in sleep and arousal

SR is the only known enzyme responsible for D-Ser synthesis in vivo (Fig. 1b)^35^, L-Ser and D-Ser could be converted reciprocally to each other by SR. Sleep defect in *shmt-es* mutant flies is consistent with a role for either D- or L-Ser, whereas the phenotypes in *srko* mutants suggest that D-Ser is important for sleep. To further distinguish between D- and L-Ser, they were separately applied to *srko* flies.

As discussed earlier, both L-Ser and D-Ser could rescue the sleep defect of *shmt-es* flies (Fig. 1f). However, only D-Ser, but not L-Ser, could rescue the sleep defect of *srko* flies (Fig. 2d). No significant sleep change was observed in *srko* flies fed with L-Ser compared to mock. By contrast, the nighttime sleep duration of *srko* was rescued by D-Ser to the level of *wt* flies fed with mock. When we examined the arousal response, we found that the arousal rate of *srko* flies was also rescued to the *wt* level by D-Ser, but not by L-Ser (Fig. 2f). These results suggest that D-Ser, but not L-Ser, is important for sleep and arousal.

### Increased sleep and decreased arousal in *daao-dko* mutants

**Fig. 3:**
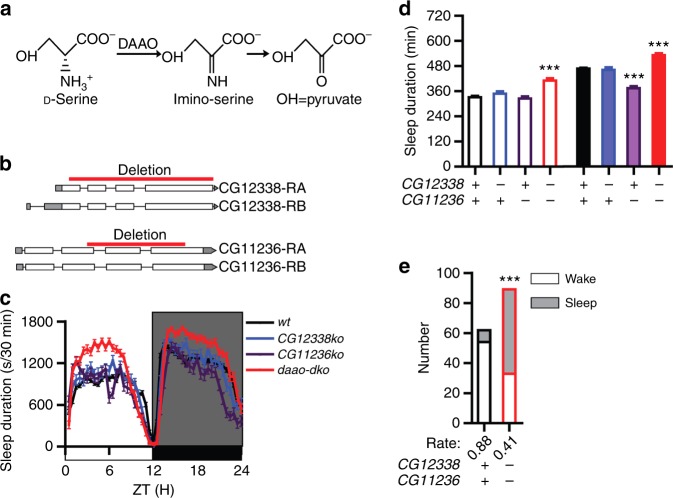
Sleep phenotype of *daao* mutants. **a** A diagram of D-Ser degradation pathway. **b** Schematic representations of *CG12338* and *CG11236* genes. The red bars indicate regions deleted in mutant flies. For *CG12338*, two transcription variants (NM_001299363, NM_136759) generate the same protein of 335aa (NP_001286292, NP_610603) and most of the coding region except the first 97 base pairs (bp) was deleted in the *CG12338* knock-out (*cg12338ko*) flies. For *CG11236*, two transcription variants (NM_135231, NM_001258985) generate two proteins of 341aa (NP_609075) and 338aa (NP_001245914), respectively. *CG11236 knock-out* (*cg11236ko*) contained the first 94aa because of deletion of 284 bp to 889 bp (NM_135231) or 284 bp to 880 bp (NM_001258985) from each transcript which resulted in frameshifts. **c** Sleep profiles of *cg12338ko* (blue) (*n* = 42), *cg11236ko* (magenta) (*n* = 46), *double knock-out* (*daao-dko*, red) (*n* = 40), and *wt* (black) (*n* = 200) flies, plotted in 30 min bins. **d** Statistical analyses. Both daytime and nighttime sleep durations were significantly increased in *daao-dko* flies, nighttime sleep duration was significantly decreased in *cg11236ko* flies. **e** Arousal rate was significantly reduced in *daao-dko* flies. Numbers of flies were plotted for *wt* (black) and *daao-dko* (red) flies. ***P < 0.001. Mann–Whitney test was used in (**d**), Fisher’s exact test was used in (**e**). Error bars represent s.e.m. Male flies were used

D-Ser is degraded by DAAOs (Fig. 3a). There are two genes encoding DAAO in *Drosophila*: *CG12338* and *CG11236*^36^. To investigate their functional significance, we generated deletion mutants for each of the gene (Fig. 3b).

When a single *daao* was interrupted, there was not much change in sleep durations: nighttime sleep duration was significantly reduced in *CG11236ko* flies, while daytime sleep duration of *CG11236ko* and daytime and nighttime sleep durations of *CG12338ko* were not different from that in the *wt* (Fig. 3c, d). In order to tell that if this is due to the redundant function of these two *daao* genes, we generated double knock-out (*daao*-*dko*) flies with both genes interrupted. We found that both daytime and nighttime sleep durations were significantly increased in *daao*-*dko* flies (Fig. 3c, d), and the arousal rate of *daao*-*dko* flies was significantly decreased (Fig. 3e). The opposite sleep and arousal phenotype of *daao*-*dko* flies to that in the *srko* flies further supports that D-Ser promotes sleep and inhibits the arousal response in *Drosophila*.

### D-Ser regulation of sleep through NMDAR1

D-Ser is a co-agonist of the NMDA receptor (NMDAR)^13,14^. There are two NMDAR subunits in *Drosophila* but only NMDAR1 contains the D-Ser binding site^37^. Pan-neuronal NMDAR1 knockdown by *elav-Gal4* driven RNA interference (RNAi) reduces sleep in *Drosophila*^22^, but it did not distinguish whether the sleep effect was caused by D-Ser or by other NMDAR1 agonists, such as glycine. Recently, a study has found that NMDAR-mediated field excitatory post-synaptic potentials (NMDA-fEPSPs) and D-Ser levels fluctuate with sleep need in mice^24^, further raising the possibility of D-Ser regulating sleep through NMDAR1, in both flies and mammals.

**Fig. 4:**
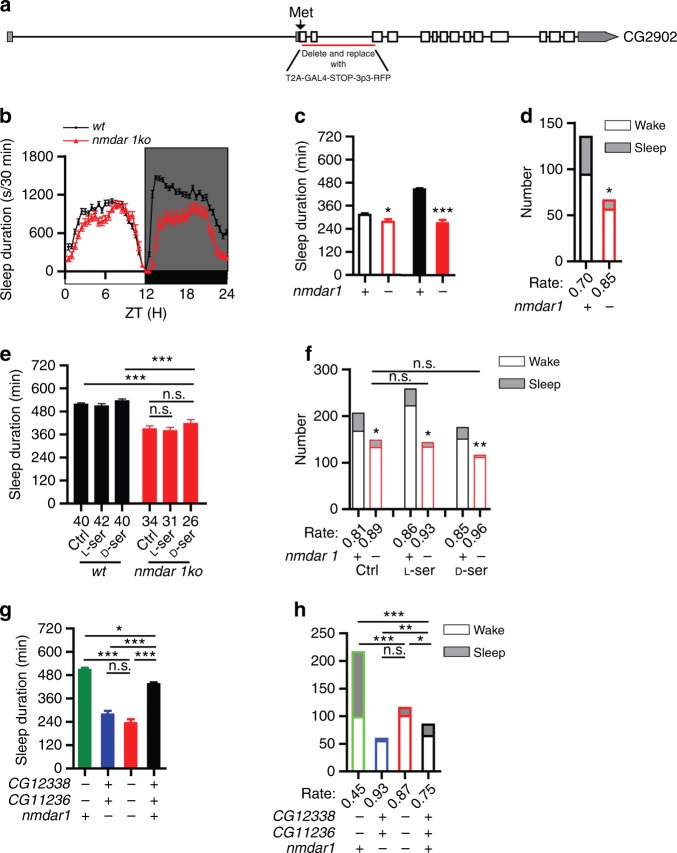
Regulation of sleep by D-Ser upstream of the NMDAR1. **a** A schematic representation of *nmdar1* gene. The single transcription variant (NM_169059) generates a protein of 997aa (NP_730940). The sequence from aa2 to 107 was deleted and replaced with T2A-Gal4-STOP-3P3-RFP in *nmdar1ko* flies. **b** Sleep profiles of *nmdar1ko* (red) (*n* = 36) and *wt* (black) (*n* = 69) flies, plotted in 30 min bins. **c** Statistical analyses. Daytime and nighttime sleep durations were significantly reduced in *nmdar1ko* flies. **d** Arousal rate of *nmdar1ko* flies (red) was significantly higher than that of *wt* (black). Numbers of flies that were aroused (filled bars) and that kept sleep (open bars) were plotted for *nmdar1ko* and *wt* flies. **e**, **f** Neither L- nor D-Ser affected the sleep duration (**e**) or the arousal rate (**f**) of *nmdar1ko* flies. Numbers below each bar represent the number of flies tested in (**e**). **g**, **h** The sleep phenotype (**g**) and arousal phenotype (**h**) of *daao-dko* flies were masked by *nmdar1ko* in triple knockout flies. Nighttime sleep durations were significantly increased in *daao-dko* (green) (*n* = 46) flies, and significantly decreased in *nmdar1ko* (blue) (*n* = 32) and triple knockout (red) (*n* = 34) flies, compared to *wt* (black) (*n* = 47) flies (**g**). Arousal rate was significantly decreased in *daao-dko* (green) flies, and significantly increased in *nmdar1ko* (blue) and triple knockout (red) flies, compared to *wt* (black) flies (**h**). ***P < 0.001, **P < 0.01, *P < 0.05, n.s. *P* > 0.05. Mann–Whitney test was used in (**c**), two-way ANOVA test with Bonferroni posttests was used in (**e**) to compare the sleep durations between *wt* and *nmdar1ko* under the same treatment, Kruskal–Wallis test with Dunn’s posttest was used in (**e**) for other statistical analyses and in (**g**). Fisher’s exact test was used in (**d**), (**f**), and (**h**). Error bars represent s.e.m. Male flies were used

To investigate whether NMDAR1 regulates sleep, we generated *nmdar1ko* by replacing the first three coding exons of *nmdar1* with *2A-Gal4-STOP* right after the start codon (Fig. 4a). Similar with *srko* flies, sleep duration was significantly decreased and arousal rate significantly increased in *nmdar1ko* flies (Fig. 4b–d).

We carried out two experiments to investigate the relation between D-Ser and NMDAR1 in sleep and arousal: pharmacological application of L-Ser and D-Ser on *nmdar1ko* flies, and generation of triple knock-out flies lacking the *nmdar1* and the two *daao* genes.

Although D-Ser rescued the sleep defect and arousal defect in *shmt-es* and *srko* flies (Figs. 1f and 2d, f), neither L-Ser nor D-Ser could affect the sleep duration and arousal rate of *nmdar1ko* mutants (Fig. 4e, f). These results support that D-Ser lies downstream of SHMT and SR but upstream of NMDAR1 in sleep regulation.

To test the epistasis relationship of *daao* genes and the *nmdar1* gene, triple knockout flies carrying all three mutations of *CG12338ko*, *CG11236ko*, and *nmdar1ko* were generated by combining *daao-dko* and *nmdar1ko* together. Triple knockout flies phenocopy *nmdar1ko* flies in sleep duration and arousal rate: sleep duration was increased and arousal rate decreased in *daao-dko* flies, while sleep duration was decreased and arousal rate increased in *nmdar1ko* and triple knockout flies (Fig. 4g, h). No significant difference was detected between the sleep duration and arousal rate of *nmdar1ko* and triple knockout flies (Fig. 4g, h). These results indicate that *nmdar1* acts downstream of *daao*.

Thus, both the pharmacological experiment and the genetic epistasis experiment support that D-Ser functions through NMDAR1 to regulate sleep in *Drosophila*.

### Expression patterns of *shmt*, *sr*, *daao*, and *nmdar1*

To examine the expression of genes participated in the synthesis, degradation, and function of D-Ser, we fused Gal4 in-framely to *shmt*, *sr*, *CG12338*, *CG11236*, and *nmdar1*, making the *shmt-KIGal4*, *SR-KIGal4*, *CG12338-KIGal4*, *CG11236-KIGal4*, and *nmdar1-KIGal4* lines (Supplementary Table 1). Then *UAS-mCD8::GFP* was driven by these lines to label the membrane of cells expressing them respectively.

**Fig. 5:**
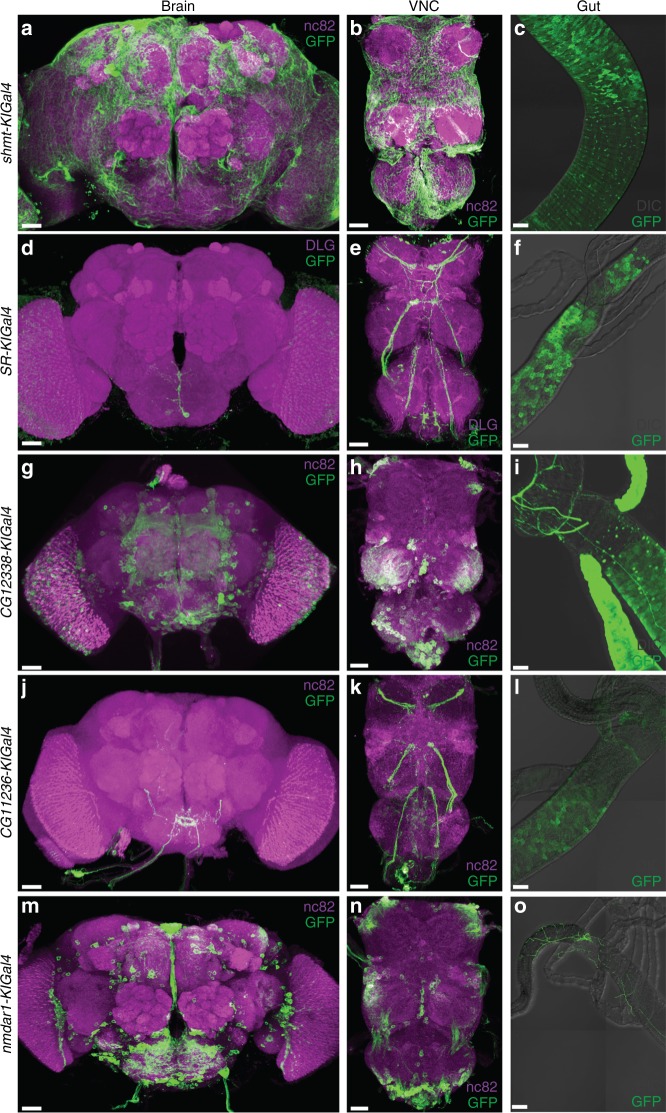
Expression patterns of *shmt*, *sr*, *daao*, and *nmdar1*. Expression patterns of *shmt-KIGal4* (**a**–**c**), *SR-KIGal4* (**d**–**f**), *CG12338-KIGal4* (**g**–**i**), *CG11236-KIGal4* (**j**–**l**), and *nmdar1-KIGal4* (**m**–**o**) labeled by mCD8::GFP in the brain (**a**, **d**, **g**, **j**, **m**), the ventral nerve cord (VNC) (**b**, **e**, **h**, **k**, **n**), and the gut (**c**, **f**, **i**, **l**, **o**). The tissues were immunostained with anti-GFP and anti-DLG in (**d**, **e**), immunostained with anti-GFP in (**c**, **f**, **i**, **l**, **o**), immunostained with anti-GFP and nc82 in other panels. Scale bars are 30 μm

We found that all five lines were expressed in the brain, the ventral nerve cord (VNC), and the gut (Fig. 5, Supplementary Fig. 5). *shmt-KIGal4* was expressed in the glia cell and neurons in the brain and the VNC (Fig. 5a, b), and in the midgut (Fig. 5c, Supplementary Fig. 5a). *SR-KIGal4* was expressed in four neurons in the subesophageal ganglion (SOG) of the brain (Fig. 5d), in four pairs of neuronal tracts projecting to the prothoracic, mesothoracic, metathoracic neuromere (PN, MN, MtN), and the abdominal center (AC) of the VNC (Fig. 5e), and in the midgut enterocytes (ECs) (Fig. 5f, Supplementary Fig. 5b).

*CG12338-KIGal4* was expressed in the MB, the PI, and the SOG of the brain (Fig. 5g), in the MN, the MtN, and the AC of the VNC (Fig. 5h), and in the midgut, the Malpighian tubules, and the neurons projecting to the hindgut (Fig. 5i, Supplementary Fig. 5c). *CG11236-KIGal4* was expressed similarly to *SR-KIGal4*: in quite a few neuron tracts projecting to the SOG of the brain (Fig. 5j), in four pairs of neuronal tracts projecting to the PN, the MN, the MtN, and the AC of the VNC (Fig. 5k), and in the midgut ECs (Fig. 5l, Supplementary Fig. 5d).

*nmdar1-KIGal4* was expressed broadly in the brain, including the PI, the SOG, the FSB, and the superior neuropils (SNP) (Fig. 5m), in the AC and the afferent neurons of the PN of the VNC (Fig. 5n), and in neurons projecting to the proventriculus, the midgut regions R1 and R5, and the hindgut (Fig. 5o, Supplementary Fig. 5e).

### Intestinal SR in sleep regulation

Given that the synthases, the oxidases, and the receptor of D-Ser were all found to be expressed in the central nervous system and the gut, we next seek to identify which part is required for D-Ser to promote sleep by reintroducing *UAS-SR* into different parts in the *srko* background.

**Fig. 6:**
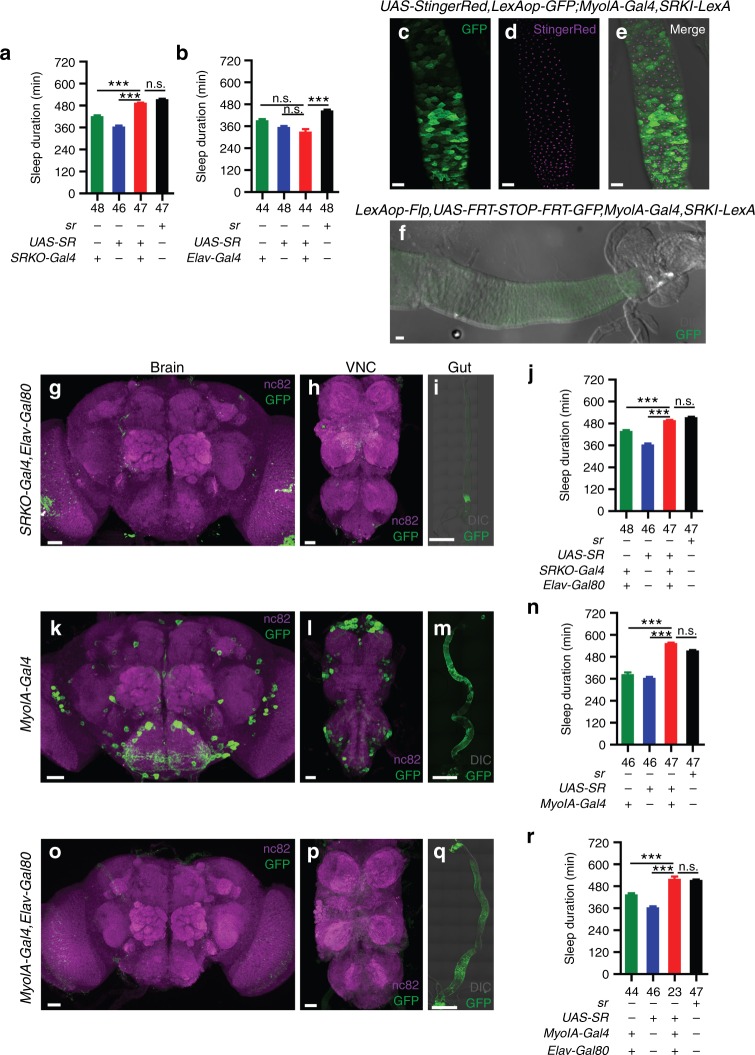
Requirement of intestinal but not neural SR in sleep regulation. **a** Reintroduction of *sr* in *sr*-expressing cells rescued the nighttime sleep defect of *sr* mutants. Nighttime sleep durations of *srko/SRKO-Gal4* (green), *srko/srko,UAS-SR* (blue), *SRKO-Gal4/srko,UAS-SR* (red), and *wt* (black) flies were plotted. **b** Reintroduction of *sr* pan-neuronally failed to rescue the sleep defect of *sr* mutants. Nighttime sleep durations of *Elav-Gal4/Y; srko/srko* (green), *srko/srko,UAS-SR* (blue), *Elav-Gal4/Y; srko/srko,UAS-SR* (red), and *wt* (black) flies were plotted. **c**–**e** *sr*-expressing cells and *MyoIA*-expressing cells overlap in the fly gut. The nuclei of *MyoIA*-expressing cells were labeled by StingerRed driven by *MyoIA-Gal4* (**d**), and the *sr*-expressing cells were labeled by GFP driven by *SRKI-LexA* (**c**). **f** Expression patterns of *LexAop-Flp, UAS-FRT-STOP-FRT-GFP, MyoIA-Gal4, SRKI-LexA* flies in the gut. Cells co-expressing *MyoIA* and *sr* were labeled with GFP. **g**–**i** *sr*-expressing non-neuronal cells were labeled by *SRKO-Gal4,Elav-Gal80* (**i**), whereas the neural cells expression was blocked (**g**, **h**). **j** Reintroduction of *sr* driven by *SRKO-Gal4,Elav-Gal80* rescued the sleep defect of *sr* mutants. Nighttime sleep durations of *srko/SRKO-Gal4,Elav-Gal80* (green), *srko/srko,UAS-SR* (blue), *SRKO-Gal4,Elav-Gal80/srko,UAS-SR* (red), and *wt* (black) flies were plotted. **k**–**m** Expression patterns of *MyoIA-Gal4* in the brain (**k**), the VNC (**l**), and the gut (**m**) labeled by mCD8::GFP. **n** Expression of *sr* in *MyoIA*-expressing cells rescued the nighttime sleep defect of *sr* mutants. Nighttime sleep durations of *srko/srko,MyoIA-Gal4* (green), *srko/srko,UAS-SR* (blue), *srko,MyoIA-Gal4/srko,UAS-SR* (red), and *wt* (black) flies were plotted. **o**–**q** *MyoIA*-expressing non-neuronal cells were labeled by *MyoIA-Gal4,Elav-Gal80* (**q**), whereas the neural cells expression was blocked (**o**, **p**). **r** Expression of *sr* driven by *MyoIA-Gal4,Elav-Gal80* rescued the sleep defect of *sr* mutants. Nighttime sleep durations of *srko/srko,MyoIA-Gal4,Elav-Gal80* (green), *srko/srko,UAS-SR* (blue), *srko,MyoIA-Gal4,Elav-Gal80/srko,UAS-SR* (red), and *wt* (black) flies were plotted. The tissues were immunostained with anti-GFP in (**c**–**f**), (**i**, **m**), and (**q**), immunostained with anti-GFP and nc82 in (**g**, **h**, **k**, **l**, **o**, **p**). Scale bars are 500 μm in (**i**, **m**, **q**), 30 μm in other panels. Numbers below each bar represent the number of flies tested. Kruskal–Wallis test with Dunn’s posttest, ***P < 0.001, n.s. *P* > 0.05. Error bars represent s.e.m. Male flies were used

The nighttime sleep duration of *sr* mutants was rescued by the reintroduction of *UAS-SR* back into *sr*-expressing cells (Fig. 6a) labeled by *SRKO-Gal4* in which *2A-Gal4-STOP* was fused to the start codon of *sr* (Supplementary Table 1). However, pan-neuronal expression of *sr* driven by *Elav-Gal4* failed to rescue the nighttime sleep duration (Fig. 6b), suggesting that *sr* does not function in neurons to promote sleep. Furthermore, we labeled non-neuronal *sr*-expressing cells by *SRKO-Gal4, Elav-Gal80*, in which neuronal expression but not intestinal expression of *SRKO-Gal4* was blocked by *Elav-Gal80* (Fig. 6g–i). Reintroduction of *sr* back into non-neuronal *sr*-expressing cells also rescued the sleep defect of *sr* mutants (Fig. 6j), suggesting that neuronal *sr* is not necessary for sleep promoting. Taken together, these results suggest that neuronal *sr* is neither sufficient nor necessary for sleep promoting, thus *sr* functions elsewhere to regulate sleep.

Because *sr* was expressed in the midgut ECs (Fig. 5f, Supplementary Fig. 5b), we used *MyoIA-Gal4* which was known to drive GFP expression in midgut ECs^38^ to test the role of intestinal SR in sleep regulation. mCD8::GFP was driven by *MyoIA-Gal4* to label *MyoIA*-expressing cells, *MyoIA-Gal4* was expressed in the midgut ECs (Fig. 6m) as well as in the neurons in the brain and the VNC (Fig. 6k, l), while no expression of *MyoIA-Gal4* was found in the genital and the internal surface of the abdominal cuticle which is covered by the fat body and the oenocytes (Supplementary Fig. 6). Colocalization of *MyoIA* and *sr* was detected in the gut by simultaneously labeling *sr*-expressing cells with GFP and the nuclei of *MyoIA*-expressing cells with StingerRed (Fig. 6c–e). We also intersected *MyoIA* and *sr* by expressing *UAS-FRT-STOP-FRT-GFP* in *MyoIA*-expressing cells, and expressing *LexAop-Flp* in *sr*-expressing cells. Thus, in cells that co-expressing *MyoIA* and *sr*, the stop cassette between the UAS and GFP was removed by the Flp recombinase, labeling the cells with GFP (Fig. 6f). Expression of *sr* in *MyoIA*-expressing cells rescued the sleep defect of *sr* mutants (Fig. 6n). We also used Elav-Gal80 to block the neuronal expression of *MyoIA-Gal4* (Fig. 6o–q), and the nighttime sleep duration of *sr* mutants was rescued by expression of *sr* in non-neuronal *MyoIA*-expressing cells (Fig. 6r). Moreover, the sleep duration was significantly decreased and sleep latency significantly increased when *sr* was knocked-down with RNAi specifically in the gut (Supplementary Fig. 7a, d) or *daao* was overexpressed specifically in the gut (Supplementary Fig. 7b, c, e, f) with *daao* cDNA driven by *MyoIA-Gal4, Elav-Gal80*. Taken together, these results support that SR expressed in intestinal cells is important for regulating sleep in *Drosophila*.

## Discussion

Our findings have revealed both a novel function for D-Ser and a novel role for intestinal cells. Results from mutations of five genes (two genes required for D-Ser synthesis, two for D-Ser degradation, and one for the D-Ser receptor), taken together with those from the pharmacological application of L- and D-Ser, support the conclusion that D-Ser plays an important role through NMDAR1 in regulating sleep in *Drosophila*. Furthermore, results from genetic rescue experiments with neuronal and intestinal drivers indicate that intestinal SR regulates sleep.

The evidence for D-Ser function in sleep is strong. Phenotypic analysis indicates that D-Ser is important for nighttime sleep and arousal. Nighttime sleep and arousal phenotypes of *shmt*, and *sr* mutants are opposite to those of *daao-dko* mutants, consistent with roles of D-Ser in increasing sleep and decreasing arousal. Further support was provided by the finding that D-Ser could rescue the sleep and arousal phenotypes in *shmt* and *sr* mutants, whereas L-Ser was unable to rescue the sleep and arousal phenotypes in *sr* mutants. In *Drosophila*, while the functional significance of D-Ser in sleep is clear, a role for L-Ser appears unlikely but cannot be completely ruled out at this point.

While NMDAR1 could affect circadian rhythm in mice^39,40^, it is surprising for a well-known excitatory receptor to promote sleep. Our results from *nmdar1* knockout flies and *sr* knockout flies provide the strongest in vivo evidence for an essential role of NMDAR1 in promoting sleep. These results are consistent with, but cleaner than, the previous RNAi results in flies^22^. NMDAR1 has recently been implicated in regulating sleep in flies^22,23^: pan-neuronal knocked down of NMDAR1 or NMDAR2 through RNAi or feeding of the NMDAR antagonist MK801 to flies reduced sleep duration^22^. So far, regional RNAi had failed to reveal specific regions where NMDAR1 regulates sleep^22^. NMDAR1 expression has been detected in the R2 ring of the EB which is important for sleep homeostasis^23^, though it remains unknown whether NMDAR1 in the R2 ring regulates sleep.

Roles for D-Ser in regulating mammalian sleep and arousal remain to be investigated. The saturation level of the glycine binding site in the NMDAR1 correlates with sleep need in mice^24^. Total serine level increased to ~487% during slow wave sleep (SWS) in the ventrolateral posterior nuclei (VLPN) of the cat thalamus^41^. D-Ser reduces sedative response induced by alcohol in flies and rodents^31,42^. A report of the effect of *daao* ablation on promoting sedative response in mice under novel environment^43^ was refuted by further analysis^44^. Because D-Ser was only increased in some but not all regions in the brain after the elimination of a single *daao*^45^, the function of D-Ser in mammalian sleep remains unclear.

Both glial and neuronal distribution of D-Ser and SR have been reported in mammals^35,46^. Early studies have detected D-Ser to be present in glia^10,47,48^. D-Ser has been considered as a major gliotransmitter^49–51^ and its release is triggered by non-NMDAR glutamate receptor^48,49^. However, SR and D-Ser were also found in neurons^47,52,53^. Both the neuronal and glial release of D-Ser have been detected^54^. Using conditional SR knocked out mice, the majority of SR (65%) and extracellular D-Ser have been suggested to be of neuronal origin^55^. Our present study with *Drosophila* using *SR-KIGal4* identified only neuronal but no glial expression of SR.

A striking finding here is that SR is not only expressed in the nervous system, but that it is expressed and functions in intestinal cells to regulate sleep in *Drosophila*. Through region-specific rescue, knock-down, and overexpression studies (Fig. 6, Supplementary Fig. 6), we found that the expression of SR in cells labeled by *MyoIA-Gal4, Elav-Gal80* is essential for sleep regulation. We have examined the expression patterns only in the CNS, the gut, the genital, the fat body, and the oenocytes, therefore, although it is most likely that SR in the intestinal cells functions to regulate sleep, functions in other organs or cells could not be completely ruled out.

This is the first time that a gene has been found to function in the intestines to regulate sleep in any animal species. Why and how intestinal SR regulates sleep remains elusive. Sleep disorders have been found to be associated with gastrointestinal and metabolism pathology in human^56^ and animals^57^. The intestine is a tissue made up of a large variety of cells^58^ that could both sense the environment and communicate with the central nervous system. In mammals and flies, crosstalk between enteroendocrine cells and neurons through neuropeptide signaling have been identified to regulate processes such as energy homeostasis and development^59,60^, and the gut microbiome has been implicated in the regulation of behaviors such as locomotion and anxiety^61–63^. We have now demonstrated an essential role for an endogenous gene in the intestine in sleep regulation. How D-Ser produced in the intestine functions through the NMDAR to regulate sleep, and whether other cells, such as glia, participate in the circuit requires further studies. Our work should stimulate further investigations of whether *sr* or other genes function in the gastrointestinal system to regulate sleep or other neuronal functions.

## Methods

### Fly lines and rearing conditions

All fly stocks were reared on standard corn meal at 25 °C and 50% humidity on a 12:12 LD schedule unless otherwise noted. *Elav-Gal4, Elav-Gal80, UAS-mCD8::GFP*, *UAS-StingerRed*, *LexAop-GFP* were from the Bloomington Stock Center. *MyoIA-Gal4* were generously provided by R. Xi (National Institute of Biological Science, Beijing). *UAS-srRNAi* (v110407) and *UAS-Dicer* (v60009) were from the Vienna *Drosophila* RNAi Center.

### Generation of transgenic, knockout, and knockin flies

We generated *UAS-SR*, *UAS-CG12338*, and *UAS-CG11236* flies by inserting the coding sequences of *CG8129-RB*, *CG12338-RA*, and *CG11236-RA* respectively into the PACU2 vector from the Jan lab at UCSF^64^, and then inserted the construct into *attp2* site. The coding sequences were amplified from the 1st strand cDNA made by the PrimeScript™ II 1st strand cDNA synthesis kit (Takara, 6210A) from total RNA of *wt* flies isolated with TRIzol reagent (Invitrogen).

We generated fly mutants with CRISPR/Cas9. A pSP6-2sNLS-spcas9 plasmid and a pMD19-T gRNA scaffold vector were obtained from Dr. R. Jiao^65^. After the sequence of single strand guide RNA (sgRNA) was designed, the corresponding DNA template was amplified from the pMD19-T gRNA scaffold vector, and then transcribed in vitro (Promega, P1320) to obtain the sgRNA. pSP6-2sNLS-spcas9 vector was linearized by restriction enzyme *XbaI* (New England BioLabs, USA, R0145), transcribed in vitro (Ambion, USA, AM1340), and added with Poly(A) (New England BioLabs, USA, M0276) to obtain spCas9 mRNA. To generate *shmt-es* mutant, we injected one single strand guide RNA (sgRNA) and a spCas9 mRNA into Canton-S (CS) embryos to generate indel induced by site-directed cleavage in *CG3011*. F2 indel flies were identified by PCR and confirmed by sequencing. Mutants with a stop codon in the coding region of *CG3011* were selected for further studies. To generate *srko*, *CG12338ko*, and *CG11236ko* flies, we injected two sgRNAs and a spCas9 mRNA into CS embryos. The target region between the two sgRNAs was deleted. F2 knock-out flies were identified by PCR and sequencing.

To obtain Gal4/LexA lines, we injected two sgRNA, spCas9 mRNA, and a donor plasmid into CS embryos. Gal4/LexA in the donor plasmid was integrated into specific sites of the genome through homologous recombination. A sgRNA and a spCas9 mRNA were used to improve the efficiency of homologous recombination. To construct the donor plasmid, two homologous arms were amplified by PCR from the fly genome using restriction enzyme-tailed PCR primers and the products were inserted into appropriate restriction sites in the pBlueScriptII vector. T2A-Gal4/LexA-loxP-3P3-RFP-loxP sequence (constructed by Bowen Deng in the Lab) was inserted between the two homologous arms. T2A-Gal4/LexA was kept in-frame with the 5′ homologous arm. 3P3-RFP was used as a selection marker after injection. F2 flies with RFP observed in the eyes was selected and confirmed by PCR and sequencing (Supplementary Table 1). Sequences of the primers used for identification of the fly lines are presented in Supplementary Table 2.

### Behavioral assays

Sleep analysis was performed in a video-based recording system. 5–8 days old flies were placed in 65 mm × 5 mm tubes containing fly food. Infrared LED lights were used to provide constant illumination, and videos were recorded by a camera with 704 × 576 resolution. Videos were taken at 5 frames/s. And 1 frame/s was extracted for fly tracing. The position of a fly was tracked by a program based on OpenCV. Briefly, flies were extracted by subtracting background which was updated for each frame in order to prevent environmental light shift. The center of a fly was calculated, the speed was defined as changes of the center from the previous frame to the current frame. Sleep was defined with more than 5 min bout of inactivity, sleep latency was defined as the time in minutes from the moment light was turned off to the onset of sleep^16,17^.

Arousal response was measured at ZT16 (4 h after lights off) under 1 s light stimulation (100–200 lux)^34^. The percentage of flies that were aroused by light stimuli from sleep was calculated as arousal rate.

Sleep deprivation was performed by placing a silica gel holder with recording tubes horizontally into a holding box. The box was rotated clock-wise or counter clock-wise and bumped to plastic stoppers under the control of a servo motor. The box was rotated continuously for 9 times during each episode, and the setup was activated every 3 min for 12 h during the night. Sleep was completely deprived as confirmed by DAM recording during deprivation^66^. Because sleep homeostasis is more significant in females than that in males^67^, we used females to examine sleep homeostasis.

For analysis of circadian rhythm, flies were treated and recorded in the same condition as the sleep assay, except that the experiments were performed in DD. Activity was measured for 5–8 days and calculated in ActogramJ^68^. The period length was calculated by Chi-square method.

### Drug treatment

L-Ser (S0035) and D-Ser (S0033) were from Tokyo Chemical Industry and mixed in the 5% sucrose and 2% agar medium. Flies were maintained on food containing 2.9 g/L L-Ser, D-Der (treatment group) or no Ser (control group) after eclosion for 3 days before being transferred to the recording tube with the same medium.

### Immunohistochemistry and confocal imaging

Flies were anesthetized and dissected in cold phosphate-buffered saline (PBS). Brains were fixed in 4% paraformaldehyde (weight/volume) for 1 h at room temperature (RT), washed in PBST (PBS containing 0.2% Triton X-100, vol/vol) for 10 min three times, blocked in PBSTS (PBS containing 2% Triton X-100, 10% normal goat serum, vol/vol) for 12 h at 4 °C, incubated with primary antibodies in the dilution buffer (PBS containing 0.25% Triton X-100, 1% normal goat serum, vol/vol) for 12 h at 4 °C, and washed with the washing buffer (PBS containing 1% Triton X-100, vol/vol, 3% NaCl, g/ml) for 10 min three times. Brains were then incubated with secondary antibodies in the dilution buffer for 12 h at 4 °C in darkness, and washed three times in the washing buffer for 10 min each. In the case of the third antibody, brains were further incubated with third antibodies at 4 °C overnight and washed. Finally, brains were mounted in Focusclear (Cell Explorer Labs, FC-101) and imaged on Zeiss LSM710 confocal microscope.

The intestines, after dissection, were fixed with 4% paraformaldehyde for 0.5 h, followed by 30 s in heptanes and methanol (1:1, vol/vol), washed with 100% methanol for 5 min twice, and washed with PBST for 10 min three times, and immunostained for GFP as described in the previous paragraph. Images were processed by Zeiss software and Imaris (bitplane) for 3D reconstruction.

Chicken anti-GFP (1:1000) (Abcam Cat# 13970; RRID:AB_300798), mouse anti-Bruchpilot (1:40) (DSHB Cat# 2314866, nc82; RRID: AB_2314866) were used as primary antibodies with AlexaFluor488 anti-chicken (1:500) (Life Technologies Cat# A11039; RRID:AB_2534096) and AlexaFluor633 anti-mouse (1:500) (Life Technologies Cat# A21052; RRID: AB_141459) being used as respective secondary antibodies. Mouse 4F3 anti-DLG (1:50) (DSHB Cat# 4F3 anti-discs large; RRID: AB_528203) was used as primary antibody with biotin-conjugated goat anti-mouse (1:200) (Invitrogen Cat# B2763; RRID: AB_2536430) being the secondary antibody and AlexaFluor635 streptavidin (1:500) (Invitrogen Cat# S32364) being the third antibody.

### Quantitative PCR

Total RNA was extracted from ~60 flies aged 5–8 days using TRIzol reagent (Invitrogen) and then reverse transcripted by the PrimeScript^TM^ RT Master Mix kit (Takara, RR036A). Quantitative PCR analysis was then performed using TransStart Top Green qPCR SuperMix kit (TransGen, AQ131-03) in the Applied Biosystems 7900HT Fast-Time PCR system. The sequences of primers used to detect *shmt* and *actin42a* (endogenous control) RNA are as follows:

shmt-F: 5′-CAGCCGTTTACAAAGACATGCA-3′

shmt-R: 5′-GAATGGCGTTGGTGATGGTT-3′

act42a-F: 5′-CTCCTACATATTTCCATAAAAGATCCAA-3′

act42a-R: 5′-GCCGACAATAGAAGGAAAAACTG-3′

### Statistics

All statistical analyses were carried out with Prism 5 (GraphPad Software). Fisher’s exact test was used to compare arousal rates. Mann–Whitney test was used to compare two columns of data. Kruskal–Wallis test followed by Dunn’s posttest was used to compare multiple columns of data. Two-way ANOVA followed by Bonferroni post-tests was used to compare drug rescue effects. Additional Mann–Whitney test was used to compare mutants with *wt* flies under different treatments. Statistical significance is denoted by asterisks: *P < 0.05, **P < 0.01, ***P < 0.001.

## Supplementary information

Supplementary Information

Reporting summary

## Data Availability

The data that support the findings of this study are available upon reasonable request.

## Code availability

Code used for tracing the fly were conducted in C++, and further analyses were conducted in Matlab. All the code are available upon reasonable request.
