# Supplementary material for: D-Serine made by serine racemase in Drosophila intestine plays a physiological role in sleep
Source: Nat Commun. 2019 May 7;10:1986. doi: 10.1038/s41467-019-09544-9 (PMC6504911; doi:10.1038/s41467-019-09544-9)
Supplement: Supplementary file 1 — Supplementary Information [file 41467_2019_9544_MOESM1_ESM.pdf]

Supplementary Information

A Novel Physiological Role in Sleep for D-Serine Made by Serine Racemase in *Drosophila* Intestine

Xihuimin Dai, Enxing Zhou *et al.*

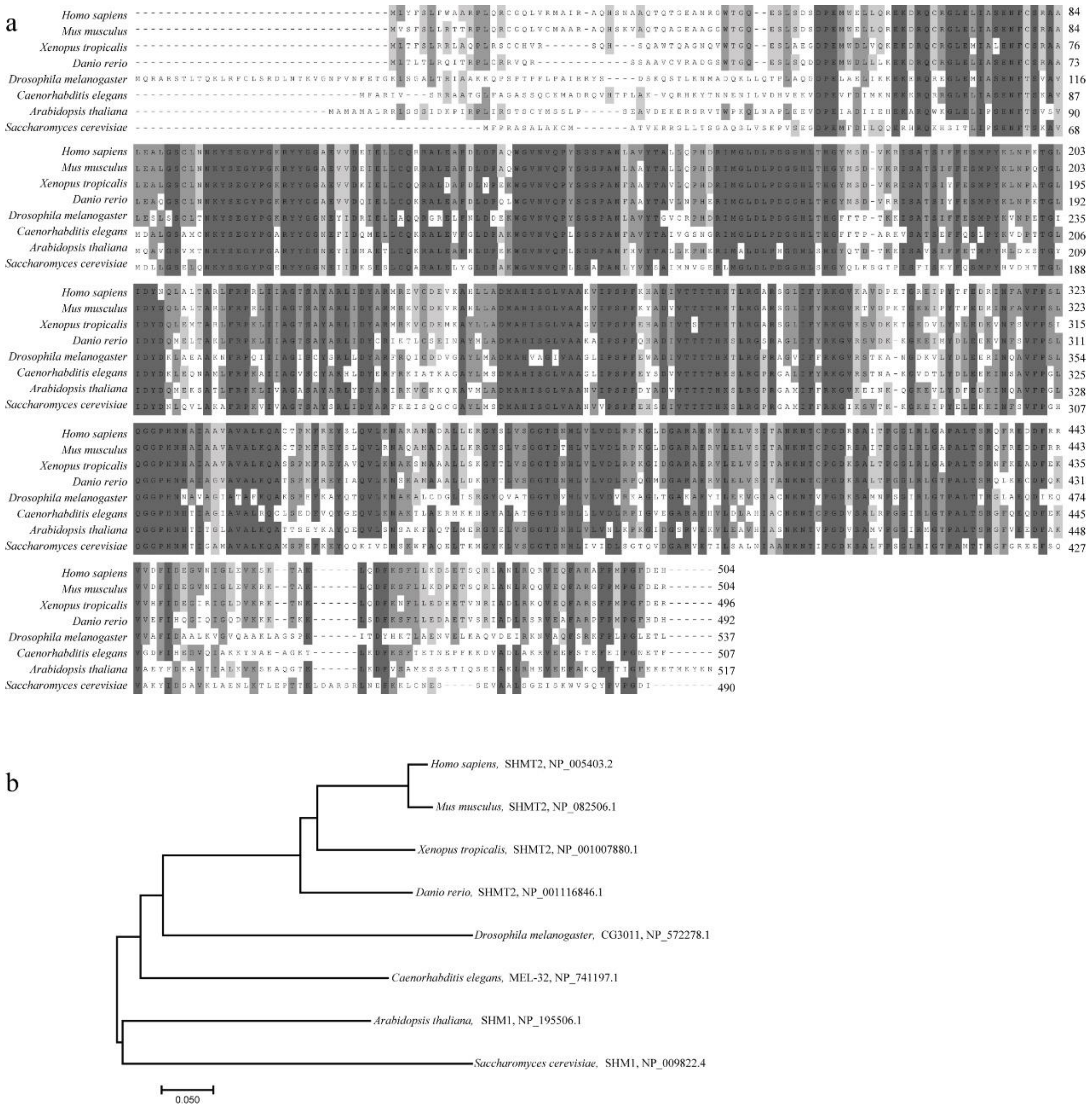
a
Homo sapiens
Mus musculus
Xenopus tropicalis
Danio rerio
Drosophila melanogaster
Caenorhabditis elegans
Arabidopsis thaliana
Saccharomyces cerevisiae
b
Homo sapiens, SHMT2, NP_005403.2
Mus musculus, SHMT2, NP_082506.1
Xenopus tropicalis, SHMT2, NP_001007880.1
Danio rerio, SHMT2, NP_001116846.1
Drosophila melanogaster, CG3011, NP_572278.1
Caenorhabditis elegans, MEL-32, NP_741197.1
Arabidopsis thaliana, SHM1, NP_195506.1
Saccharomyces cerevisiae, SHM1, NP_009822.4
0.050

Supplementary Figure 1. Alignment of SHMT proteins. a, Alignment of the predicted amino acid (aa) sequence of *Drosophila CG3011* isoform PA (GenBank number NP_572278) with SHMT from other species using Clustal Omega. Identical aa residues are shaded in black, and similar residues in gray. **b,** A phylogenetic tree of SHMT proteins. The scale bar represents 5% aa substitutions per residue. *Drosophila* SHMT protein is 57% identical to *Homo sapiens* SHMT2 (NP_005403), 56% identical to *Mus musculus* SHMT2 (NP_082506), 58% identical to *Xenopus tropicalis* SHMT2 (NP_001007880), 56% identical to *Danio rerio* SHMT2 (NP_001116846), 56% identical to *Caenorhabditis elegans* MEL-32 (NP_741197), 51% identical to *Arabidopsis thaliana* SHM1 (NP_195506), 49% identical to *Saccharomyces cerevisiae* SHM1 (NP_009822).

a

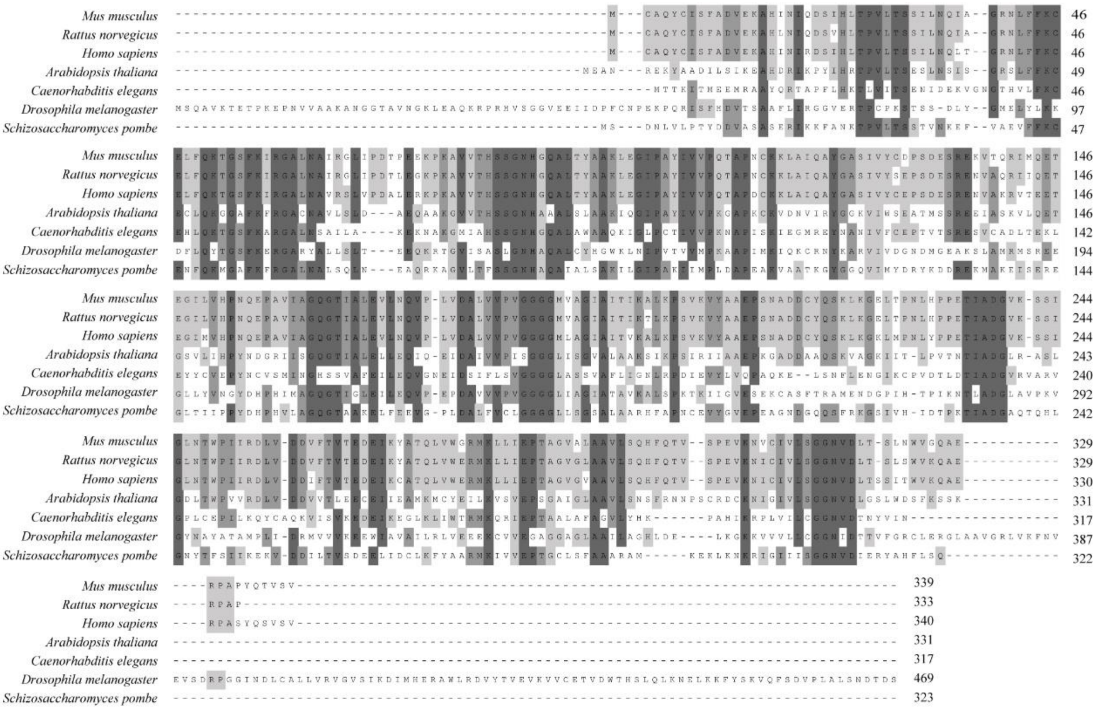
Mus musculus
Rattus norvegicus
Homo sapiens
Arabidopsis thaliana
Caenorhabditis elegans
Drosophila melanogaster
Schizosaccharomyces pombe
46
46
46
49
46
97
47
146
146
146
146
142
194
144
244
244
244
243
240
292
242
329
329
330
331
317
387
322
339
333
340
331
317
469
323

b

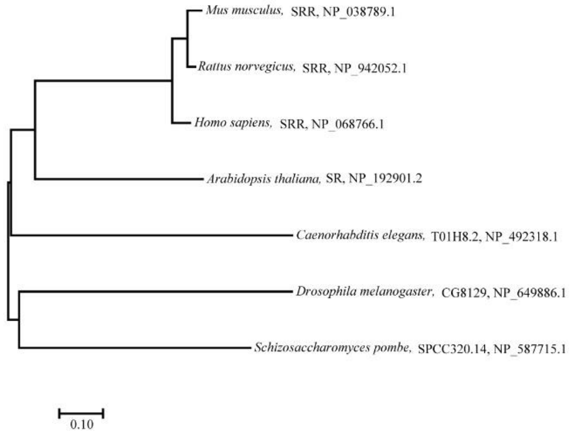
Mus musculus, SRR, NP_038789.1
Rattus norvegicus, SRR, NP_942052.1
Homo sapiens, SRR, NP_068766.1
Arabidopsis thaliana, SR, NP_192901.2
Caenorhabditis elegans, T01H8.2, NP_492318.1
Drosophila melanogaster, CG8129, NP_649886.1
Schizosaccharomyces pombe, SPCC320.14, NP_587715.1
0.10

Supplementary Figure 2. Alignment of SR Proteins. a, Alignment of the predicted amino acid sequence of *Drosophila* CG8129 isoform PB (GenBank number NP_649886) with SRs from other species using Clustal Omega. Identical amino acid residues are in black, and similar residues in gray. **b,** A phylogenetic tree of SRs. The scale bar represents 10% amino acid substitutions per residue. *Drosophila* SR is 35% identical to *Homo sapiens* SRR (NP_068766), 33% identical to *Mus musculus* SRR (NP_038789), 34% identical to *Rattus norvegicus* SRR (NP_942052), 28% identical to *Caenorhabditis elegans* T01H8.2 (NP_492318), 34% identical to *Arabidopsis thaliana* SR (NP_192901), 32% identical to *Schizosaccharomyces pombe* SPCC320.14 (NP_587715).

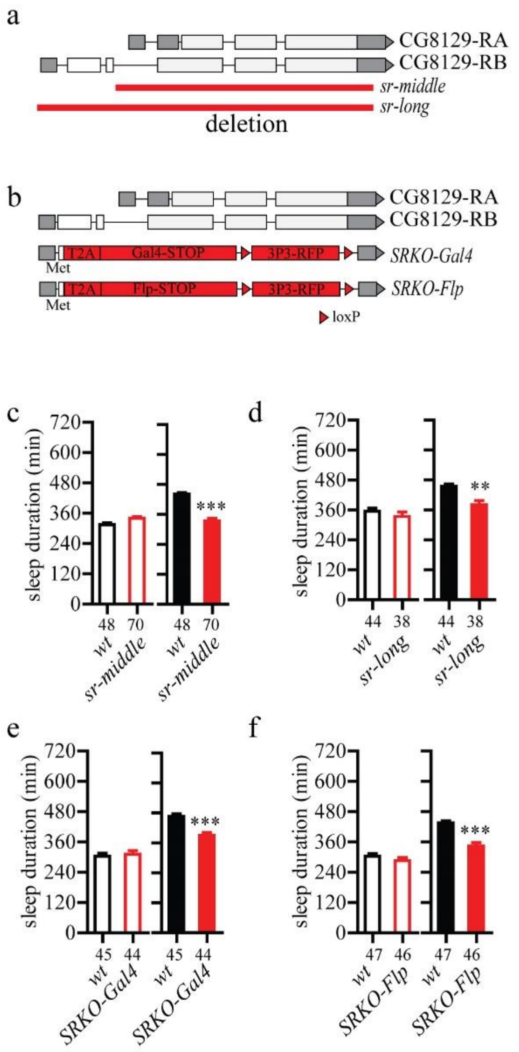

Supplementary Figure 3. Sleep Phenotypes of *sr* Mutants. a-b, Schematic representations of four different *sr* mutants, including two deletion mutants **(a)** and two insertion mutants **(b)**. **c-f,** Nighttime sleep duration was significantly decreased in four different sr mutants: *sr-middle* **(c)**, *sr-long* **(d)**, *SRKO-Gal4* **(e)**, and *SRKO-Flp* **(f)**. Open bars denote daytime sleep, filled bars denote nighttime sleep. Numbers below each bar represent the number of flies tested. Mann Whitney test, *** $P<0.001$, ** $P<0.01$. Error bars represent s.e.m. Male flies were used.

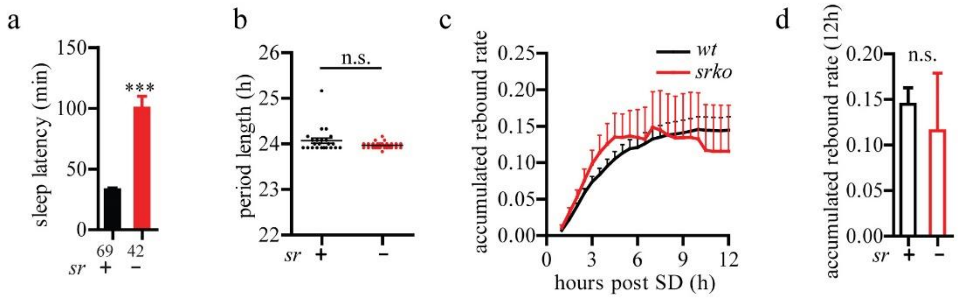

Supplementary Figure 4. Sleep and Circadian Phenotypes of *srko* Flies. a, Sleep latency of *srko* (red) (n=42) flies was significantly longer compared to wt (black) (n=69) flies. **b,** Circadian period length of *srko* (red) (n=26) had no significant difference with *wt* (black) (n=23) flies. Circadian rhythms were calculated in ActogramJ[1]. **c,** Accumulated rebound curves of *srko* (red) (n=52) and *wt* (black) (n=77) flies after one night of sleep deprivation. **d,** Statistical analysis of rebound rate at 12 h post-sleep deprivation. No significant difference was detected between *srko* and *wt* flies. Mann Whitney test, n.s. $P>0.05$. Error bars represent s.e.m. Male flies were used in **(a-b)**. Female flies were used in **(c-d)**.

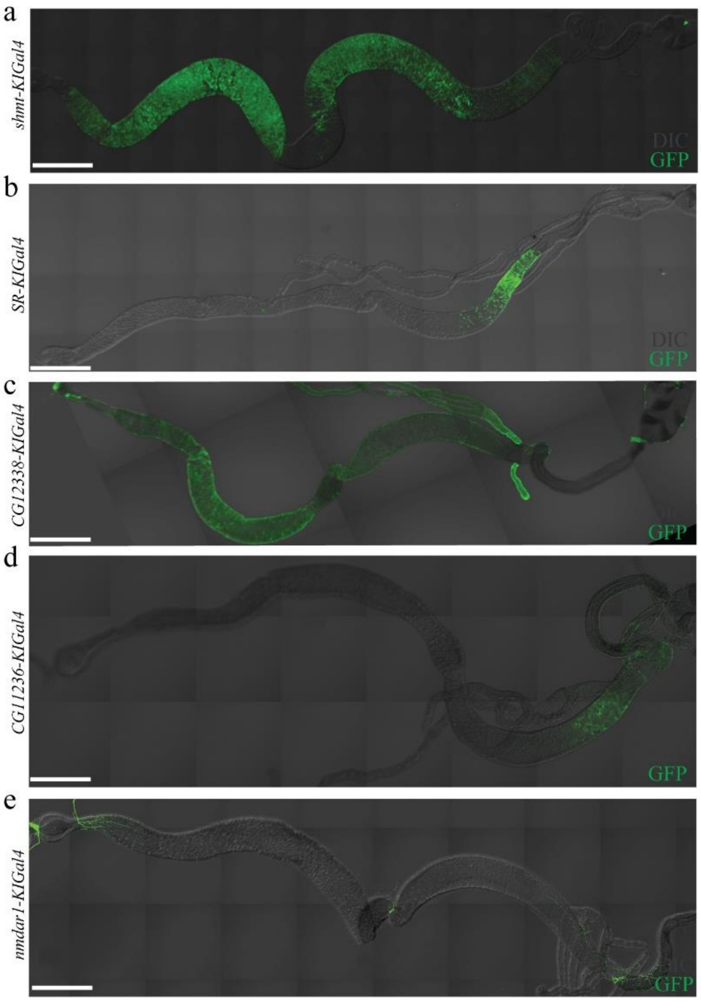

Supplementary Figure 5. Expression Patterns of *shmt*, *sr*, *daao* and *nmdar1* in the Gut. a-e, Expression patterns of *shmt-KIGal4* **(a)**, *SR-KIGal4* **(b)**, *CG12338-KIGal4* **(c)**, *CG11236-KIGal4* **(d)**, and *nmdar1-KIGal4* **(e)** labeled by mCD8::GFP in the gut. The guts were immunostained with an anti-GFP antibody. Scale bars are 500μm.

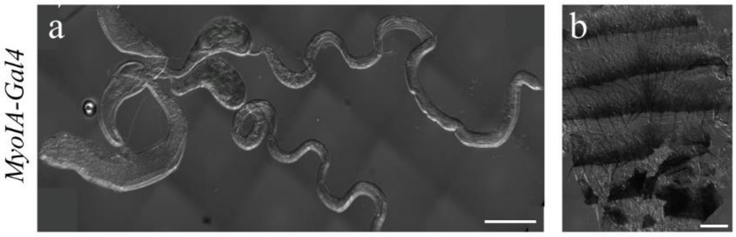

Supplementary Figure 6. No Expression of *MyoIA-Gal4* was Detected in the Genital (a) and the Internal Abdominal Cuticle Surface (b). Expression patterns of *MyoIA-Gal4* were labeled by mCD8::GFP. The tissues were immunostained with anti-GFP. Scale bars are 200μm.

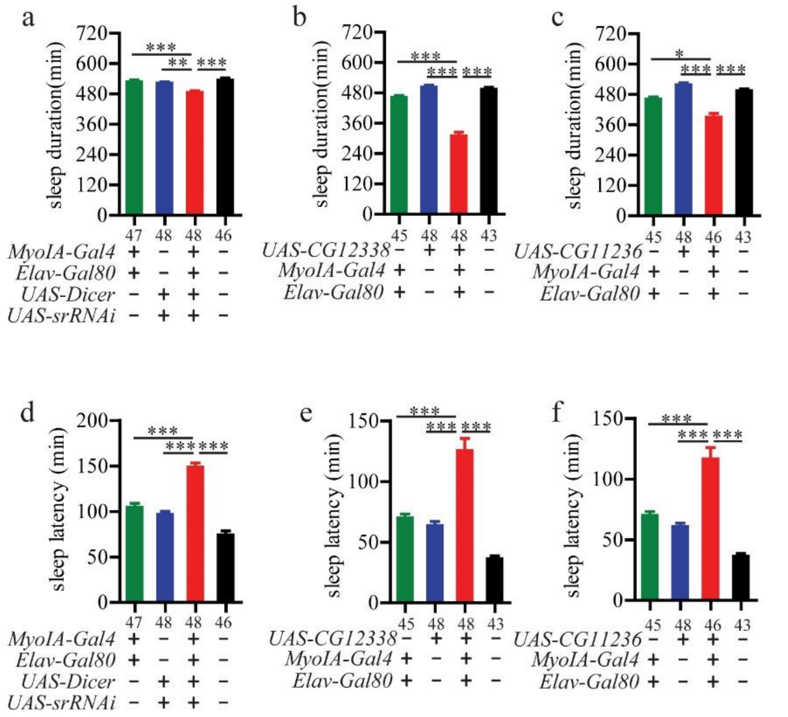

Supplementary Figure 7. Sleep Phenotypes of Gut-specific D-Ser Downregulation. **a-c,** Sleep duration was significantly decreased in gut-specific *sr* knockdown **(a)** and gut-specific *daao* overexpression **(b-c)**. **d-f,** Sleep latencies were significantly increased in gut-specific *sr* knockdown **(d)** and gut-specific *daao* overexpression **(e-f)**. Numbers below each bar represent the number of flies tested. Mann Whitney test, *** $P<0.001$, ** $P<0.01$, * $P<0.05$. Error bars represent s.e.m. Male flies were used.

Supplementary Table 1. List of genotypes of generated fly lines. Targeted region is based on FB2018_02, released Apr 3, 2018 by FlyBase. Mutated sequences are in red characters.

Constructed line	targeted region	manipulation
srko	3R: 9366409..9367626	deleted
CG12338ko	2R:10729640..10730764	deleted
CG11236ko	2L:6803390..6804121	deleted
nmdar1ko	3R: 5514628..5516383	replaced with 2A-Gal4-STOP
shmt-KIGal4	X:5913046..5913199	replaced with 2A-Gal4-STOP
SR-KIGal4	3R: 9367596..9367646	replaced with 2A-Gal4-STOP
SR-KILexA	3R: 9367596..9367646	replaced with 2A-LexA-STOP
CG12338-KIGal4	2R:10729128..10729641	replaced with 2A-Gal4-STOP
CG11236-KIGal4	2L:6804256..6804630	replaced with 2A-Gal4-STOP
nmdar1-KIGal4	3R: 5508989..5509789	replaced with 2A-Gal4-STOP
SRKO-Gal4	3R: 9365491..9367646	replaced with 2A-Gal4-STOP
sr-middle	3R: 9365859..9367626	deleted
sr-long	3R: 9365158..9367626	deleted
SRKO-Flp	3R: 9365491..9367646	replaced with 2A-Flp-STOP
	**Genotype**	**Protein Sequence**
wildtype	ATGCAGCGGGCGCGCTCTACACTGACACAAAAGC TTCGGTTTTGCCTTAGTCGGGACCTGAACACCAA AGTTGGCAACCCGGTTAAC…	MQRARSTLTQKLRFCLSRDLNTKVGNPV
shmt-es	ATGCAGCGGGCGCGCTCTACACTGACACAAAAGC TTCGGTTTTGCCTTAGTCGGGACCTGAACACCAG TTGGCAACCCGGTTAA…	MQRARSTLTQKLRFCLSRDLNTSWQPG*

Supplementary Table 2. List of the Sequences of Primers Used for Identification of Generated Lines.

Constructed line	Forward primer	Reverse primer	Length
srko	SR-F: 5'-aatgggcgtgtcttcgaact-3'	SR-R:5'-acgtccgaggacttgacaac-3'	2140bp
sr-middle			1590bp
sr-long			889bp
CG12338ko	12338-F: 5'-ggagagcggatttgataagagc-3'	12338-R: 5'-atgcaacgcaccaactgttc-3'	867bp
CG11236ko	11236-F: 5'-aacgaaaggtccgagagttt-3'	11236-R: 5'-atgcttgcattgacccaatc-3'	950bp
nmdar1ko	NMDAR1KO-F: 5'-ccctcatcttgcctctt-3'	GAL4-R: 5'-tctgtgacggcatcttt-3'	2961bp
shmt-KIGal4	SHMTKI-F: 5'-ttccgaagtgcgtcag-3'		2537bp
SR-KIGal4	SRKI-F: 5'-aacgcatatccttccatgac-3'		2422bp
CG12338-KIGal4	12338KI-F: 5'-ttgattatccctgtcacgtc-3'		2387bp
CG11236-KIGal4	11236KI-F: 5'-gtcaaagggtcatgaaggat-3'		2980bp
nmdar1-KIGal4	NMDAR1KI-F: 5'-aaagtgtagcgaagcagagg-3'		2598bp
SRKO-Gal4	SRKO-F: 5'-tggcagcattgtggagaacagt-3'		2170bp
SRKO-Flp	SRKO-F: 5'-tggcagcattgtggagaacagt-3'	Flp-R: 5'-tgccgcagttgatgaat-3'	2448bp
SR-KILexA	SRKI-F: 5'-aacgcatatccttccatgac-3'	LexAR: 5'-tcctggcaacgactacctg-3'	2511bp

Supplementary References:

1. Schmid, B., Helfrich-Förster, C. & Yoshii, T. A new ImageJ plug-in ‘ActogramJ’ for chronobiological analyses. *J. Biol. Rhythms* **26,** 464–467 (2011).
