# Supplementary material for: D-Serine made by serine racemase in Drosophila intestine plays a physiological role in sleep
Source: Nat Commun. 2019 May 7;10:1986. doi: 10.1038/s41467-019-09544-9 (PMC6504911; doi:10.1038/s41467-019-09544-9)
Supplement: Supplementary file 2 — Reporting summary [file 41467_2019_9544_MOESM2_ESM.pdf]

nature research

Corresponding author(s): Yi Rao

Last updated by author(s): Mar 4, 2019

Reporting Summary

Nature Research wishes to improve the reproducibility of the work that we publish. This form provides structure for consistency and transparency in reporting. For further information on Nature Research policies, see Authors & Referees and the Editorial Policy Checklist.

Statistics

For all statistical analyses, confirm that the following items are present in the figure legend, table legend, main text, or Methods section.

n/a	Confirmed	
☐	☒	The exact sample size (*n*) for each experimental group/condition, given as a discrete number and unit of measurement
☐	☒	A statement on whether measurements were taken from distinct samples or whether the same sample was measured repeatedly
☐	☒	The statistical test(s) used AND whether they are one- or two-sided<br>*Only common tests should be described solely by name; describe more complex techniques in the Methods section.*
☒	☐	A description of all covariates tested
☒	☐	A description of any assumptions or corrections, such as tests of normality and adjustment for multiple comparisons
☒	☐	A full description of the statistical parameters including central tendency (e.g. means) or other basic estimates (e.g. regression coefficient) AND variation (e.g. standard deviation) or associated estimates of uncertainty (e.g. confidence intervals)
☒	☐	For null hypothesis testing, the test statistic (e.g. *F*, *t*, *r*) with confidence intervals, effect sizes, degrees of freedom and *P* value noted<br>*Give P values as exact values whenever suitable.*
☒	☐	For Bayesian analysis, information on the choice of priors and Markov chain Monte Carlo settings
☒	☐	For hierarchical and complex designs, identification of the appropriate level for tests and full reporting of outcomes
☒	☐	Estimates of effect sizes (e.g. Cohen's *d*, Pearson's *r*), indicating how they were calculated

Our web collection on statistics for biologists contains articles on many of the points above.

Software and code

Policy information about availability of computer code

Data collection	ZEN 2012 Version 11.0.4.190
Data analysis	IMARIS 7.2.3 Build 23216 for Win32; ImageJ 1.50d; ActogramJ; Prism 5 Version 5.01; Matlab R2010a; SingleTracer_v2.0.2.1

For manuscripts utilizing custom algorithms or software that are central to the research but not yet described in published literature, software must be made available to editors/reviewers. We strongly encourage code deposition in a community repository (e.g. GitHub). See the Nature Research guidelines for submitting code & software for further information.

Data

Policy information about availability of data

All manuscripts must include a data availability statement. This statement should provide the following information, where applicable:

- Accession codes, unique identifiers, or web links for publicly available datasets
- A list of figures that have associated raw data
- A description of any restrictions on data availability

All the data is available under request.

Field-specific reporting

Please select the one below that is the best fit for your research. If you are not sure, read the appropriate sections before making your selection.

☒ Life sciences ☐ Behavioural & social sciences ☐ Ecological, evolutionary & environmental sciences

For a reference copy of the document with all sections, see nature.com/documents/nr-reporting-summary-flat.pdf

Life sciences study design

All studies must disclose on these points even when the disclosure is negative.

Sample size	We used ~48 flies of each genotype for one experiment, this sample size is big enough to get reproducible results.
Data exclusions	Dead flies were excluded at the end of the experiments.
Replication	All the experiments were repeated 3 times.
Randomization	Flies used in the experiments were picked randomly.
Blinding	The tracing and analyzing were processed by softwares, the investigators were blind to group allocation.

Reporting for specific materials, systems and methods

We require information from authors about some types of materials, experimental systems and methods used in many studies. Here, indicate whether each material, system or method listed is relevant to your study. If you are not sure if a list item applies to your research, read the appropriate section before selecting a response.

Materials & experimental systems

n/a	Involved in the study	
☐	☒	Antibodies
☒	☐	Eukaryotic cell lines
☒	☐	Palaeontology
☐	☒	Animals and other organisms
☒	☐	Human research participants
☒	☐	Clinical data

Methods

n/a	Involved in the study	
☒	☐	ChIP-seq
☒	☐	Flow cytometry
☒	☐	MRI-based neuroimaging

Antibodies

Antibodies used	chicken anti-GFP (Abcam Cat#13970; RRID:AB_300798), mouse anti-Bruchpilot (DSHB Cat# 2314866, nc82;RRID: AB_2314866), AlexaFluor488 anti-chicken (Life technologies Cat# A11039; RRID:AB_2534096), AlexaFluor633 anti-mouse (Life technologies Cat#A21052; RRID: AB_141459), mouse 4F3 anti-DLG (DSHB Cat# 4F3 anti-discs large; RRID: AB_528203), biotin-conjugated goat anti-mouse (Invitrogen Cat# B2763; RRID: AB_2536430), Alexa fluor 635 streptavidin (1:500) (Invitrogen Cat# S32364)
Validation	chicken anti-GFP (applicable for immunofluorescence in flies), mouse anti-Bruchpilot (applicable for immunofluorescence in flies), mouse 4F3 anti-DLG (applicable for immunofluorescence in flies).

Animals and other organisms

Policy information about studies involving animals; ARRIVE guidelines recommended for reporting animal research

Laboratory animals	Drosophila melanogaster, isolated Canton-S, unmated female and male, 5~8 days old.
Wild animals	No wild animals is involved.
Field-collected samples	No field-collected samples is involved.
Ethics oversight	No ethical approval or guidance was required for flies.

Note that full information on the approval of the study protocol must also be provided in the manuscript.
